# Golimumab for Polyarticular Juvenile Idiopathic Arthritis and Psoriatic Arthritis: Pharmacologic and Clinical Considerations

**DOI:** 10.3390/life13071601

**Published:** 2023-07-21

**Authors:** Sydney Moore McIntosh, Christian Kerut, Payton P. Hollenshead, Dorothy H. Askins, Kasra Mansourian, Zachary R. Palowsky, Varsha Allampalli, Shahab Ahmadzadeh, Sahar Shekoohi, Alan D. Kaye

**Affiliations:** 1 School of Medicine, Louisiana State University Health Sciences Center at Shreveport, Shreveport, LA 71103, USA; 2 School of Medicine, Louisiana State University Health Sciences Center at New Orleans, New Orleans, LA 71102, USA; 3 Department of Anesthesiology, Tulane University, New Orleans, LA 70112, USA; 4 School of Medicine, Tulane University, New Orleans, LA 70112, USA; 5 Department of Anesthesiology, Louisiana State University Health Sciences Center at Shreveport, Shreveport, LA 71103, USA; 6 Department of Pharmacology, Toxicology, and Neurosciences, Louisiana State University Health Sciences Center at Shreveport, Shreveport, LA 71103, USA

**Keywords:** golimumab, juvenile idiopathic arthritis, psoriatic arthritis, autoimmune inflammatory diseases, pediatric arthropathies

## Abstract

Psoriatic arthritis is a chronic debilitating autoimmune condition, and when diagnosed in patients before the age of eighteen, it is considered pediatric polyarticular juvenile idiopathic arthritis. Monoarticular or polyarticular psoriatic arthritis can be distinguished from other arthropathies by its unique cutaneous manifestations. With numerous treatments already in clinical practice, there are numerous options for treatment. The current literature indicates an elevated level of tumor necrosis factor is present in the epidermis of patients with psoriatic arthritis when compared with the general population. For this reason, anti-tumor necrosis factor therapies have become a hallmark option for psoriatic arthritis patients. Golimumab, a human monoclonal antibody tumor necrosis factor-alpha (TNF-a) receptor antagonist, was chosen as the focus therapy for this investigation. The mechanism of action behind anti-tumor necrosis factor-alpha blockers involves the binding of human TNF-a soluble and transmembrane proteins to competitively inhibit TNF-a from binding to its cellular receptors. The present investigation evaluated current treatment options available for both juvenile- and adult-onset psoriatic arthritis and compared them with the efficacy seen with golimumab use. Pediatric patients included children ages 2–17, while adult populations included adults 18–83 years old. The Food and Drug Administration has approved golimumab for the treatment of rheumatoid arthritis, psoriatic arthritis, ankylosing spondylitis, ulcerative colitis, and polyarticular juvenile idiopathic arthritis. The results of four different studies reporting on the therapeutic effects and adverse events of golimumab use in psoriatic arthritis, juvenile psoriatic arthritis, juvenile idiopathic arthritis, and juvenile polyarticular arthritis were used for comparison. The meta-analysis referenced studies including children ages 2–17 with no reference mentioning children less than age 2. Based on the results of each study, it can be concluded that golimumab, a human monoclonal antibody that prevents the activation of cellular inflammatory reactions when it binds to the TNF-*a* receptor, is an effective option for patients with active psoriatic arthritis and psoriatic juvenile idiopathic arthritis and for patients who are no longer responding to their current treatment with adalimumab. Each study also reported minimal adverse events associated with golimumab use, and the drug can be safely used in the pediatric population.

## 1. Introduction

Juvenile idiopathic arthritis is an umbrella term used to describe different inflammatory arthropathies that are diagnosed in children under the age of sixteen with symptoms lasting at least six weeks [1,2,3]. Psoriatic arthritis differs from other arthropathies, particularly in its cutaneous involvement. The disease can be monoarticular or polyarticular, but a few distinguishing characteristics include radiographic findings of the “pencil-in-cup deformity” and scaly, white skin lesions that often bleed when scratched. The radiographic findings are a result of periosteal erosion of distal interphalangeal joints, and the characteristic rash is an autoimmune attack against epidermal cells. Because psoriatic arthritis can present with a variety of symptoms, the disease is often not diagnosed until later in progression. The pathogenesis of monoarticular or polyarticular PsA is multi-factorial, with both environmental risk factors and genetic components having been identified. This allows for multiple therapeutic options consisting of different mechanisms of action to be used in disease management.

Because these conditions are progressive and usually lifelong, an important prognostic factor is early treatment initiation. Several drug classes such as non-steroidal anti-inflammatory drugs (NSAIDs), systemic and intraarticular glucocorticoids, and non-biologic and biologic disease-modifying antirheumatic drugs (DMARDs) are routinely used with varying results among patients [3]. Glucocorticoids and NSAIDs display a similar mechanism of action, in that both can be used to blunt the immune system’s release of inflammatory cytokines and decrease the number of inflammatory cells recruited to the joint spaces. A popular DMARD, methotrexate, exerts its anti-inflammatory effects by inhibiting the enzyme dihydrofolate reductase, which halts cellular DNA synthesis. Biologic DMARDs use monoclonal antibodies to target specific cellular receptors, thereby preventing further inflammatory response progression. Anti-tumor necrosis factor agents are a first-line therapy for many autoimmune conditions. By inhibiting tissue necrosis factor (TNF), T cell activation is blunted, causing a decrease in cytokine release and inflammatory cell reactions, but this effect also causes undesirable immunosuppression and an increased risk for infections.

Golimumab is a human monoclonal antibody that inhibits endogenous TNF-a from interacting with its respective receptors by binding human TNF-a soluble and transmembrane structure proteins [1]. By inhibiting TNF-a from binding to its receptor sites, the pro-inflammatory cytokine effects of TNF-a involved in inflammation, autoimmunity, and malignancy can be prevented, as the activation of these cytokine effects is dependent on TNF-a signaling [1]. As with all anti-TNF-a therapies, golimumab increases patients’ risk of upper respiratory tract infections, sinusitis, bronchitis, viral infections, and fungal infections as a result of their systemic immunosuppressive effects [1]. Because golimumab is commonly administered subcutaneously, injection site reactions such as pain, irritation, and erythema are commonly observed.

With new advancements in therapeutic options, physicians are left with the task of choosing the best algorithm for selecting therapy options based on each patient’s unique symptom and severity profiles. As with any medicine, the benefits and risks must be weighed carefully. The American College of Rheumatology has established guidelines to simplify the treatment selection process; however, in the development of new therapies such as golimumab, these guidelines risk becoming outdated if new data studies are routinely reviewed [3]. Golimumab is a human monoclonal antibody that prevents the activation of cellular inflammatory reactions when it binds to the TNF-*a* receptor [2]. This study reviewed the efficacy and safety of golimumab therapy compared with the existing first-line DMARDs available for the treatment of psoriatic arthritis, with careful consideration taken in evaluating the results seen in psoriatic juvenile idiopathic arthritis (PsJIA) versus psoriatic arthritis (PsA) in the adult population.

## 2. Psoriatic Juvenile Idiopathic Arthritis

Psoriatic juvenile idiopathic arthritis (psJIA) is a subtype of JIA that is characterized by arthritis and psoriasis. International League of Associations for Rheumatology (ILAR) criteria also include arthritis and at least two of the following findings: nail pitting or onycholysis; dactylitis; and psoriasis in a first-degree relative, excluding other causes of joint pain [4,5]. PsJIA is a chronic autoimmune disease that affects children and adolescents, and it accounts for 5–8% of all JIA cases [6,7] with a total calculated incidence of roughly three per million [6]. In this section, we will discuss the epidemiology, symptoms, and pathogenesis of psJIA.

PsJIA peaks occur bimodally and are broken down into two subgroups based on age. The early-onset group typically presents between ages 2 and 3 with a female predominance, ANA positivity, and chronic uveitis, mostly affecting the joints of the wrists and small joints of the hands and feet [8]. This is in comparison with the older subset, which typically presents between ages 8 and 12, where the patients are typically male, have HLA-B27 positivity, and have features of spondyloarthritis (SpA), such as axial pain and enthesitis [7,8]. Both subgroups typically present with dactylitis and psoriatic changes.

PsJIA can present with a variety of clinical manifestations depending on the number of joints inflamed. Most often, joint involvement occurs years before psoriasis and can mimic other forms of JIA [4]. The extent of joint involvement in arthritis can range from small-joint arthritis that affects both sides equally to large-joint involvement in the lower extremities that is asymmetrical and may develop into polyarthritis resembling rheumatoid arthritis. Notably, the presence of distal interphalangeal (DIP) joint involvement is highly suggestive of psJIA [5]. Dactylitis is another hallmark feature of psJIA, which presents as a sausage-like swelling of the fingers or toes resulting from MCP, PIP, and DIP joint involvement. Enthesitis, sacroiliitis, and spondylitis can also occur in psJIA. Psoriatic plaques often appear on the extensor surfaces of joints, hairy skin, the umbilicus, and the perineum. Nail dystrophy, onycholysis, and pitting are also common in psJIA [5]. In addition, ANA is present in a significant proportion of patients with psJIA, and HLA-B27 is present in about 30% of patients [9]. The diagnosis of psJIA is made based on clinical criteria, and laboratory tests are not specific to the disease.

Although the exact cause of psJIA is unknown, it has a complex etiology that involves both genetic and environmental factors. Several genetic loci have been shown to be associated with psJIA, including HLA-B27 (older subset) and HLA-DR5 (younger subset) [8]. Alterations in both the adaptive and innate immune systems are also thought to play a key role. JPsA is considered an autoimmune disease, and environmental triggers such as infections, trauma, antibiotics, etc., can lead to synovial inflammation [6]. Furthermore, faulty B cell tolerance has been proposed as a cause, as the autoantibodies antinuclear antibody, rheumatoid factor, and anti-citrullinated protein antibodies are produced by B cells [6]. Moreover, disturbances in the gut microbiome have been found to play a role in the pathogenesis of JIA, especially in those in the older subset [6,8].

## 3. Psoriatic Arthritis

Psoriatic arthritis (PsA) is an inflammatory spondyloarthropathy present in 30% of patients with psoriasis [1] and 0.1–1% of the general population [10,11]. Skin psoriasis (erythematous plaques/papules with silver-white scaling) precedes musculoskeletal symptoms in >70% of patients with PsA [12]. At musculoskeletal symptom onset, PsA often presents as an asymmetric oligoarthritis (<4 joints affected), with the hands, feet, and spine being the most involved [10]. As the disease progresses, more joints can be involved, leading to polyarthritis in the late disease [10]. Joint pain and tenderness are often aggravated by rest and relieved with exercise. In many patients, this condition can mimic rheumatoid arthritis, presenting with >30 min of morning stiffness. Laboratory evaluation can help distinguish between these two conditions. Anti-citrullinated peptide and rheumatoid factor are negative in 95% of patients with PsA, although the presence of these cannot exclude disease [10]. Rheumatoid arthritis can be further distinguished because it has symmetric, proximal joint involvement rather than the asymmetric, distal joint involvement that is present in >50% of patients with PsA [10]. Other associated symptoms include enthesitis (inflammation of tendon insertion sites), dactylitis (swelling of the fingers and toes: “sausage digits”), tenosynovitis (tendon inflammation), sacroiliitis, and nail involvement (brittle nails, nail pitting, onycholysis) [10]. Common X-ray findings in patients with PsA include distal interphalangeal (DIP) joints with a “pencil in cup” deformity and evidence of asymmetric erosions and ossification [13].

Many cases of psoriatic arthritis go undiagnosed, which is likely due to variability in patient presentation, a lack of effective screening modalities, or a lack of diagnostic criteria [11,13]. There are currently no established diagnostic criteria, but there are several classification criteria used for research purposes. The most widely accepted is the CASPAR (Classification Criteria for Psoriatic Arthritis) system. Following these guidelines, a patient must have an inflammatory articular disease (joint, spine, tendon) with at least three points from these criteria: current psoriasis (two points) family/personal history of psoriasis (one point); the presence of psoriatic nail findings such as onycholysis, pitting, and hyperkeratosis (one point); a negative rheumatoid factor (one point); a personal history of or current dactylitis (one point); or radiographic evidence of juxta-articular new bone formations (one point) [10,12,14,15]. The initial study in 2006 on the CASPAR system showed a 91.4% sensitivity and a 98.7% specificity for the diagnosis of PsA [10,11,14,15]. While the criteria are not diagnostic and were designed for clinical trial usage, they can be used for guidance in the clinical setting.

The pathophysiology of psoriatic arthritis is not well understood, but there are clear genetic and environmental components involved in the progression of the disease. Increases in the production of IL-23, whether due to genetic mutations, infections, or mechanical stress, can lead to chronic inflammatory changes in PsA [10,11,12,16]. IL-23 stimulates T cells to produce IL-17, IL-22, and TNF-a, which, in turn, cause inflammation, bone loss, and osteogenesis [10,16]. While these cytokines are mainly produced by CD4+ helper T cells, type 3 innate lymphoid T cells, and gamma delta T cells, CD8+ T cells are important in the pathogenesis of PsA, as evidenced by an association with major histocompatibility (MHC) class I alleles (specifically, HLA-B08, HLA-B27, HLA-B38, and HLA-B39) and the oligoclonal expansion of CD8+ T cells in arthritic joints [10,12,16].

## 4. Anti-Tumor Necrosis Factor Monoclonal Antibodies

TNF is a pro-inflammatory cytokine produced by both immune and non-immune cells to produce various biological effects [17,18]. The binding of TNF to its receptor activates numerous signaling pathways, leading to the production of transcription factors, caspases, and proteases and causing protein kinases to produce various biological effects [17,18]. There are two distinct receptors for TNF (TNFR1 and TNFR2), each with unique signaling pathways and biological responses. Tumor necrosis factor receptor 1 (TNFR1) is found in all cells (excluding erythrocytes) and induces apoptosis and acute inflammation [17,18,19]. Mutations in TNFR1 have been found to be associated with some autoimmune conditions, including ankylosing spondylitis, primary biliary cirrhosis, and multiple sclerosis [17]. Tumor necrosis factor receptor 2 (TNFR2) is expressed in lymphocytes, endothelial cells, and glial cells and is responsible for the transcriptional activation of genes that defend against pathogens, produce inflammation, induce cell proliferation and survival, and initiate apoptosis [17,18,19]. The mutation of the TNFR2 receptor has been found to be associated with some chronic inflammatory conditions, including systemic lupus erythematosus, familial rheumatoid arthritis, and ulcerative colitis [17].

TNF was initially researched as an inhibitor of tumorigenesis because of its ability to induce hemorrhagic necrosis in tumors and apoptosis [17]. Phase II clinical trials in 1989 using recombinant TNF to treat malignancy were unsuccessful and resulted in disease progression and a large side effect profile. Additional studies in 1987–1989 discovered that all joints with active RA produce pro-inflammatory cytokines, suggesting continuous unregulated production rather than transient expressions expected from mechanical or environmental stress [18]. Further evaluations of these inflammatory cascades found that TNF was a convergence point for the inflammatory cascade, pinpointing a therapeutic target for the treatment of numerous chronic inflammatory conditions.

There are currently five anti-TNF monoclonal antibodies that are FDA-confirmed for the treatment of chronic autoimmune and inflammatory conditions: etanercept, infliximab, adalimumab, certolizumab, and golimumab. All competitively inhibit TNF binding to its receptor, but they differ in pharmacokinetic and pharmacodynamic properties [20]. These variances result in changes in clinical efficacy and indications for use [20]. Major indications for the use of TNF inhibitors include inflammatory arthritis (RA, PsA, AS), inflammatory bowel disease (UC, Crohn’s), and inflammatory skin conditions (psoriasis) [20]. TNF inhibitors have been proven to increase functional ability, decrease hospitalizations and surgeries, reduce the risk of co-morbid atherosclerotic disease, and decrease bone destruction and remodeling [20]. The black box warnings for the use of all TNF inhibitors include an increased risk of serious infections (i.e., tuberculosis, invasive fungal infections, and bacterial infections with opportunistic pathogens), as well as lymphoma and other malignancies, specifically, the rare hepatosplenic T cell lymphoma [20]. Children and adolescents ages 6–17 are more likely to develop lymphomas and malignancies than children between the ages of 2 and 6 and adults over 18 [20]. These guide the main contraindications to drug use, which include congestive heart failure, hypersensitivity to drug contents, sepsis, the risk of sepsis, and active infections [20]. Before initiating TNF-a inhibitor therapies such as golimumab, patients should be administered the protein-derived derivative (PPD) test for tuberculosis, have their varicella titers checked, and undergo hepatitis B and C serologic studies [21]. Patients receiving concurrent treatments with immunomodulators and corticosteroids should also receive prophylaxis for *Pneumocystis jiroveci*, an opportunistic form of pneumonia [21]. Examples of commonly used immunomodulators include azathioprine, 6-mercaptopurine, methotrexate, leflunomide, and cyclosporine. Acute infusion reactions were noted in 15% (168/1000) of children ages 3–17, mean age 13.5, being treated with infliximab, and delayed hypersensitivity reactions (arthralgias, joint edema, fever, rash) occurred in up to 8% [21]. While only 3.3% of these patients suffered serious infections, such as sepsis, meningitis, pneumonia, opportunistic fungal infections, cutaneous tinea infections, abscesses, and herpes zoster or varicella, the risk of serious infections was higher in malnourished patients and patients receiving combination immunomodulatory therapy [21]. The most concerning long-term adverse event associated with TNF-a inhibitors is the development of hepatosplenic T cell lymphoma (HSTCL); however, it must be noted that in each reported case the patient was also being treated with thiopurines [21].

## 5. Safety and Efficacy of Golimumab in Rheumatologic Diseases

Over the past few years, extensive studies have been performed to investigate the safety and efficacy of IV and subcutaneous (sc) golimumab in adults ages 18–83 [22,23,24,25]. The GO-AFTER trial was one of the earliest studies examining the efficacy of golimumab as a treatment for active rheumatoid arthritis. This was a multicenter, randomized, double-blind, placebo-controlled, phase III clinical trial that enrolled 461 patients with active rheumatoid arthritis to receive 50 mg of sc golimumab, 100 mg of sc golimumab, or a placebo. In total, 35% of patients on 50 mg of golimumab and 38% of patients on 100 mg of golimumab achieved ACR20 at week 14, while only 18% of placebo-treated patients reached ACR20. Over 16 weeks, serious adverse events were seen in 5% of 50 mg golimumab-treated patients, 3% of 100 mg golimumab-treated patients, and 7% of placebo-treated patients [26]. Additional trials further examined the efficacy of golimumab as a treatment for other arthropathies. Notably, Kavanaugh et al. conducted the GO VIBRANT trial. This was a randomized, double-blind, placebo-controlled, phase III clinical trial. In total, 480 patients (239 controls, 241 on golimumab) with active psoriatic arthritis were randomly assigned to experimental groups to receive a placebo or 2 mg/kg of IV golimumab at weeks 0 and 4, and every 8 weeks after that. The results were analyzed using the American College of Rheumatology ≥ 20%, 50%, and 70% improvement criteria (ACR20/50/70). Evaluations at week 14 found that a greater percentage of golimumab patients reached ACR20/50/70 improvement criteria. Radiological progression was assessed using the Psoriasis Area and Severity Index ≥ 75% (PASI75) comparing baseline measurements to weeks 14 and 24 in the total modified Sharp/van der Heijede score (SHS). Experimental groups showed a greater mean change at week 14 and week 24 compared with the placebo group. Patients ≥ 18 with PsA for ≥6 months were included in the GO VIBRANT trial, with reported adverse effects including pleomorphic adenoma, myocardial infarction, pneumonia, abnormal liver function test result, neuritis, drug-induced liver injury (MTX-induced toxic hepatitis), and pustular psoriasis [24]. However, there was no remarkable difference in the number of adverse events between either group [24].

Over 52 weeks using a crossover design, the GO VIBRANT trial examined the long-term safety and efficacy of IV golimumab. Patients in the placebo group were crossed over to the golimumab treatment at week 24, with injections every 8 weeks until week 52. At week 52, the crossover group reached the same ACR20/50/70 improvements as the original golimumab group [23]. Further analysis of the GO VIBRANT trial examined health-related quality of life and work productivity by examining the change from baseline in the EuroQol-5 dimension-5 level (EQ-5d-5L) index and the Work Limitations Questionnaire (WLQ). Evaluations at week 24 found marked improvements in the golimumab group versus the placebo. Re-examinations at week 52 found the crossover group showed a change from baseline similar to the original golimumab group [23]. This crossover study showed a rapid and sustained improvement in patients with psoriatic arthritis treated with IV golimumab, with no new safety risks.

Similar crossover studies have examined the role of IV golimumab in ankylosing spondylitis (GO-ALIVE) and rheumatoid arthritis (GO-FURTHER). An additional methotrexate treatment was required in the rheumatoid arthritis study and accepted in the ankylosing spondylitis and psoriatic arthritis trials. A large study pooled the safety results of all three clinical trials and found that, while IV golimumab had a similar safety profile to other TNF inhibitors, cotreatment with methotrexate was associated with increased alanine transaminase levels and an increased incidence of serious infections [22]. The GO-VIVA trial used 127 patients between 2 and 8 years old with active polyarticular course-JIA despite ≥2 months of methotrexate treatment. In total, 84%, 80%, 70%, and 47% of patients treated with IV golimumab 80 mg/m^2^ reached JIA ACR 30, 50, 70, and 90, respectively. GO-VIVA made no reference to use in children less than two. Serious infections were reported in 6% of patients [26]. These results indicate no increase in safety risks for IV golimumab and a promising role as an effective therapy for rheumatoid arthritis, psoriatic arthritis, and ankylosing spondylitis.

A 2020 phase III, randomized, placebo-controlled trial investigated the effects of IV golimumab on patients with active psoriatic arthritis (PsA). The study found that patients receiving golimumab, as compared with a placebo, had continued improvements in both joint arthritis and skin disease after 1 year. Measurements were made using the American College of Rheumatology (ACR) ≥ 20%, 50%, and 70% improvement criteria (ACR20/50/70), as well as the Psoriasis Area and Severity Index ≥ 75% criteria (PASI75). Radiographic progression was assessed using the PsA-modified Sharp/van der Heijde score (SHS). Overall, these data suggest that golimumab improves skin and joint outcomes in patients with active PsA and that the safety profile was like that of other anti-tumor necrosis factor (TNF) agents [25]. A 2021 phase III, open-label, single-arm, international study evaluated the pharmacokinetics and safety of IV golimumab in children. ACR improvement criteria were noted. Steady-state trough concentrations and AUCs were similar to those of adult patients [26]. A 2021 retrospective study investigated the efficacy of golimumab in treating JIA patients with uveitis who stopped responding to adalimumab, the drug of choice in treating uveitis. The study found that golimumab was successful in treating all eight patients who had stopped responding to adalimumab. However, the two patients who never responded to adalimumab also did not respond to golimumab. Overall, this small study demonstrated that golimumab can be effective in treating patients who lose responsiveness to adalimumab for uveitis. A 2018 randomized, double-blind, placebo-controlled withdrawal trial explored the safety and efficacy of subcutaneous golimumab in children with active polyarticular-course juvenile idiopathic arthritis (polyJIA). The study found that, although golimumab showed significant improvement in ACR30/50/70/90, the primary endpoints, JIA flare rates, and remission were not met, as they showed similar rates to the placebo (Table 1) [23,27,28,29].

## 6. A Retrospective Systemic Literature Review and Network Meta-Analysis

**Figure 1:**
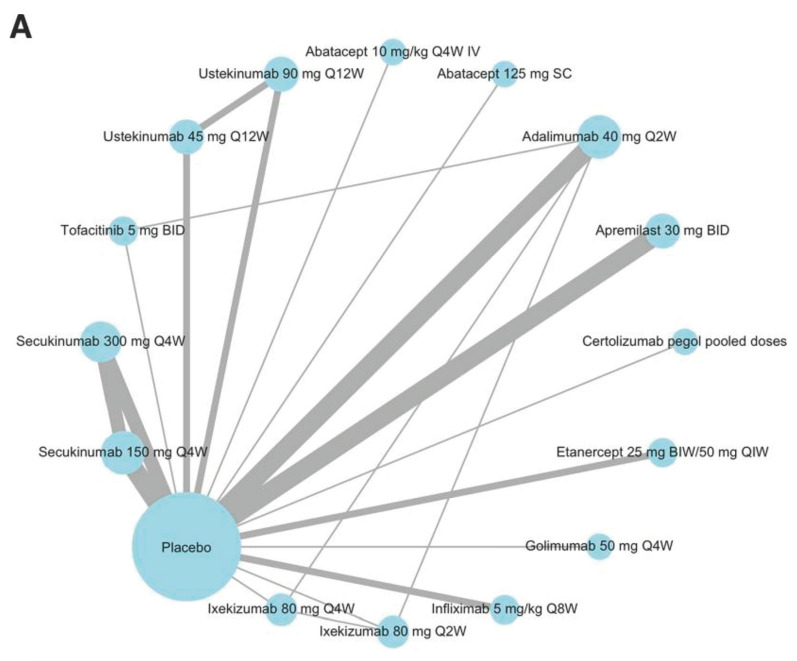

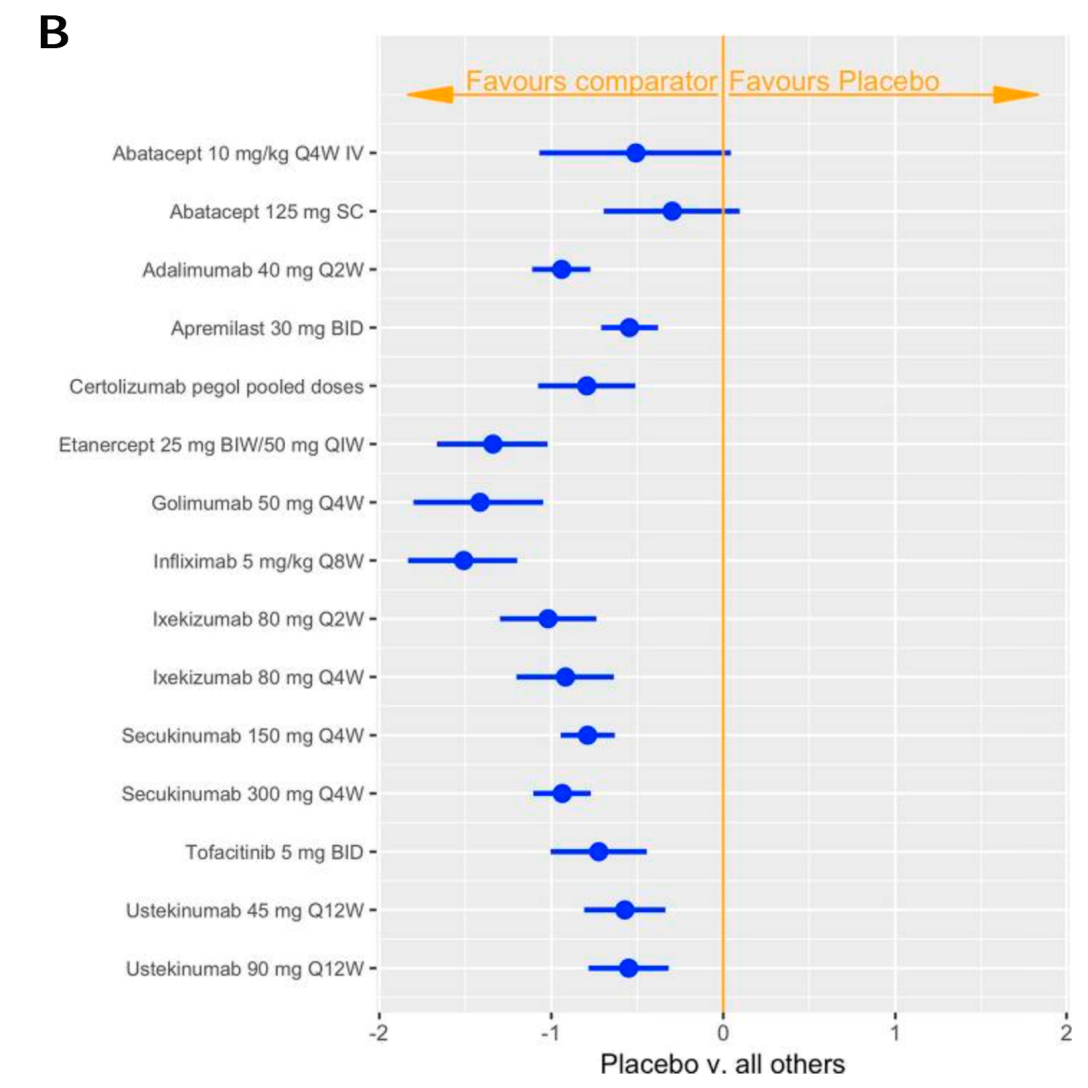
Network diagram [30] The network diagram (**A**) and forest plot (**B**) were configured based on the American College of Rheumatology (ACR) standard scale to illustrate treatment responses after 12–16 weeks of use in patients with active PsA who had never received prior treatment with a biologic DMARD. This figure was adapted with permission from RMD Open 2020;6:e001117. doi: 10.1136/rmdopen-2019-001117 [30].

A retrospective systemic literature review and network meta-analysis published in 2020 by the *British Medical Journal* compared the safety and efficacy of fifteen different biologic DMARDs in patients with psoriatic arthritis [30]. Figure 1A,B were created by the authors of “Efficacy and Safety of Biologics in PSORIATIC ARTHRITIS: A Systematic Literature Review and Network Meta-Analysis” and used as a reference for this study. The patient responses were compared with a placebo group of active PsA patients who were not treated with a biologic DMARD. The line thickness in Figure 1A corresponds to the total number of studies used for comparison that involved this therapy or that compared the therapy to an additional treatment or a placebo (Figure 1). The circle diameter corresponds to the total number of studies that involved the respective therapy. Figure 1B lists each therapy, including dosage with the route and frequency of administration. For Figure 1B, a negative value on the ACR standard normal scale indicates a more favorable response from the treatment group, with a positive value favoring the response from the placebo. The dark blue lines represent value ranges, with the blue dots emphasizing the mean (Figure 1).

The objective of the systemic literature review was to compare efficacies and side effects in drugs currently approved as PsA treatments and determine which option provides the greatest therapeutic benefit with the least number of adverse effects. TNF-a inhibitors (adalimumab, etanercept, infliximab, golimumab, and certolizumab pegol), interleukin antagonists (ustekinumab, secukinumab, and ixekizumab), and the immune suppressor abatacept were chosen for comparison, as each therapy has been approved for use in the treatment of PsA. Based on the retrospective study findings, infliximab demonstrated the highest number of responses, with golimumab having the second highest, followed by etanercept. It was concluded from the ACR scoring that treatment responses to infliximab, golimumab, and etanercept were statistically significant when compared with the placebo. Those three therapies were also deemed more effective than the other DMARDs included in the study [31]. Additional findings from the study showed no statistical increase or decrease in the chance of treatment-emergent adverse events for any of the different treatment groups when compared with the risk to patients in the placebo group [31].

## 7. Conclusions

This study focused on golimumab therapy with respect to two specific subtypes of arthritis: psoriatic polyarticular idiopathic juvenile arthritis and psoriatic arthritis. Both arthropathies are lifelong conditions, and the goal of treatment is to prevent the patients’ immune systems from forming antibodies against nonpathological antigens. Traditional therapies involve the use of various combinations of NSAIDs, DMARDs, biological agents, and glucocorticoids. While many algorithms have been developed to guide clinicians on how to treat psoriatic arthritis and its different subtypes, this review focused on golimumab’s reported safety and efficacy profile compared with other commonly used TNF-a inhibitors.

As a human monoclonal TNF-a antibody, golimumab has robust anti-inflammatory effects with a direct target in the inflammatory pathway. This made golimumab a strong candidate for the treatment of psoriatic arthritis and psoriatic polyarticular idiopathic juvenile arthritis. Golimumab was initially approved by the FDA as a treatment for rheumatoid arthritis, psoriatic arthritis, and ankylosing spondylitis in 2009 following the GO-AFTER trial. This approved the use of golimumab as a dual therapy in combination with methotrexate for adults with active psoriatic arthritis. Additional clinical trials (GO-VIBRANT, GO-ALIVE, GO-FURTHER) have shown the effectiveness of golimumab as a treatment option for psoriatic arthritis and ankylosing spondylitis as well. Randomized control trials have found that golimumab can provide patients with sustained symptom improvement when compared with a placebo.

The review noted a few special considerations for golimumab use in addition to its current indications. Golimumab can be initiated in children between ages 6 and 17 with polyarticular idiopathic juvenile arthritis who are suffering from uveitis that has become unresponsive to adalimumab therapy [29]. The GO-VIVA trial first examined the safety and efficacy of IV golimumab in patients between 2 and <8 years old. They showed that IV golimumab functioned as a successful treatment option for polyarticular course juvenile idiopathic arthritis (pc-JIA) with a low safety risk profile. Golimumab, when administered with methotrexate, demonstrated improved symptoms for pediatric patients with persistent ps-JIA who had previously shown no improvement with methotrexate monotherapy [28].

As with any treatment, the safety risks should be considered along with the patient’s lifetime risk and the severity of disease-associated symptoms. The Food and Drug Administration published a report on 3,130,267 total adverse events secondary to TNF-a inhibitor use between 2003 and 2010 [21]. The report included 91 cases of T cell non-Hodgkin lymphoma (NHL), with HSTCL being the most common type [21]. The risk of T cell NHL was found to be higher in patients receiving simultaneous treatment with TNF-a inhibitors and thiopurines (95% confidence interval, 4.98–354.09; *p* < 0.0001) [21]. This result was additionally compared with the risk associated with thiopurine monotherapy (95% confidence interval, 8.32–945.38; *p* < 0.0001) and TNF-a inhibitor monotherapy (95% confidence interval, 0.13–10.61; *p* = 1.0) [21]. The study concluded that patients receiving combination therapy (anti-TNF-a and thiopurine) are at an increased risk of developing T cell NHL compared with the less significant increased risk from thiopurine monotherapy, but TNF-a inhibitor monotherapy did not affect patient risk [21]. A study by Galloway et al. found that the risk of serious infection in rheumatoid arthritis patients treated with TNF-a inhibitors was highest in the first 6 months and then decreased over time [31]. Pooled studies on golimumab use have found it to have a similar safety profile to other FDA-approved TNF-a inhibitors [24].

To maximize safety during golimumab therapy, pediatric patients (ages 2–17) should undergo serologic testing for tuberculosis, hepatitis, and varicella–zoster virus prior to treatment initiation. Prophylactically treating patients for *Pneumocystis jiroveci,* routinely following serum drug levels, and monitoring for signs of infection will further decrease the risk of serious and opportunistic infection development. No unique adverse events were recorded for golimumab, meaning that the expected adverse events remain the same among all drugs classified as TNF-a inhibitors. The same effect can also be found when comparing disease improvement and responses in golimumab compared with other TNF-a inhibitors. Golimumab should be used in patients with psoriatic polyarticular idiopathic juvenile arthritis and psoriatic arthritis, with treatment responses expected to mirror the efficacy demonstrated by older TNF-a inhibitor therapies. Based on this report, clinicians should continue to select TNF-a inhibitor therapy based on patient-centered factors such as cost, availability, and pre-existing comorbidities with similar adverse events and efficacies among various formulations.

## Figures and Tables

**Table 1:** Clinical safety and efficacy systemic review.

Author (Year)	Groups Studied and Intervention	Results and Findings	Conclusions
Study 1: Husni [23]	Adults ≥ 18 with active PsA for ≥ 6 months were given either 2 mg/kg IV golimumab or placebo at weeks 0 and 4 and every 8 weeks.	A greater percentage of patients in the treatment group reached ACR20, ACR50, and ACR70. Radiographic progression was also significantly improved.	Golimumab was found to be significantly effective in treating patients with active PsA after 1 year. Although there were increased AEs, they were like those of other anti-TNF agents.
Study 2: Ruperto [27]	Active pc-JIA, ages 2–17, 80 mg/m^2^ golimumab. Weeks 0 and 4 and weeks 8 through 52, with methotrexate after week 28.	JIA ACR 30, 50, 70, 90 response rates for 84%, 80%, 70%, and 47% at week 28 and through to week 52; 6% with serious infections, including one death caused by sepsis.	Golimumab was effective in treating pediatric patients with pc-JIA. Serious AEs (infections) occurred in 6%., with one death.
Study 3: Lanz [28]	Ten (seventeen eyes) females ages 7–21 with active JIA-associated uveitis refractory to adalimumab received golimumab.	Eight patients with loss of response all responded to golimumab. The 2 initial non-responders did not respond to golimumab.	Golimumab is therapeutic in patients with loss of response to adalimumab, but those not responding to adalimumab did not respond to golimumab.
Study 4: Brunner [29]	In total, 173 active polyJIA patients ages 2–17 were treated with golimumab or a placebo.	After 48 weeks, there was no difference in the number of JIA flareups and clinical remission between golimumab and placebo. Golimumab was safe and tolerated well.	Golimumab resulted in significant improvement in patients with active polyJIA; however, primary endpoints were not met.

## Data Availability

Data sharing is not applicable to this article as no datasets were generated or analyzed during the current study.
